# Development and impact of virtual reality-based training for the radial forearm free flap: A multi-center prospective feasibility study

**DOI:** 10.1016/j.jpra.2025.10.024

**Published:** 2025-10-24

**Authors:** Ameen Mahmood, Clement Yong Jing Cheng, Ahmed Salih, Fay Fathima Imitaz Fareed, Princewill Ukpeh, Leena Abdalla, Andrew Gan, Kai Jian Chin, Jason Ha, Florence Chang Jia Xuan, Srija Gullapalli, Krish Gupta, Keshav Krishnan, Andrei Balan, Punn Tannirandorn, Nicole Salgado Fernandez, Iihan Ali, Anoushka Samanta, Cheuk Ying Kyleen Kiew, Joshua Lee, Manisha Teji, Abdulwali Yasini, Ayushi Gianchandani, Ameer Khamise, Afan Ali, Jagtar Dhanda

**Affiliations:** a Imperial College London, London, United Kingdom; b University College London, London, United Kingdom; c University of Buckingham, Buckingham, United Kingdom; d Barts and the London School of Medicine and Dentistry, London, United Kingdom; e University of Birmingham, Birmingham, United Kingdom; f University of Bristol, Bristol, United Kingdom; g Cardiff University, Cardiff, United Kingdom; h University of Leeds, Leeds, United Kingdom; i University of Warwick, Coventry, United Kingdom; j King’s College London, London, United Kingdom; k University of Central Lancashire, Preston, United Kingdom; l Brighton and Sussex Medical School, Brighton, United Kingdom; m Queen Victoria Hospital, East Grinstead, United Kingdom

**Keywords:** Virtual reality, Surgical education, Radial forearm free flap, Plastic surgery training, Simulation-based learning, VRiMS

## Abstract

**Introduction:**

Surgical education faces growing challenges due to reduced theatre access, variable supervision and limited procedural exposure, particularly for complex reconstructive operations such as the radial forearm free flap (RFFF). Virtual reality (VR) offers an opportunity to deliver immersive, standardized surgical training unconstrained by geography or theatre availability. This study evaluates the effectiveness of a VR-based teaching intervention in improving procedural confidence and anatomical understanding of the RFFF.

**Methods:**

A prospective multicenter feasibility study was conducted across 10 UK medical schools and one NHS trust. Participants completed a 60-minute workshop including a 360° VR simulation of the RFFF procedure and a VR anatomical exploration session. Pre- and post-workshop surveys assessed procedural confidence, anatomical understanding and user experience using validated Likert-scale tools.

**Results:**

141 participants completed both pre- and post-workshop assessments. The majority were undergraduate medical students (90.8 %), of whom 93.8 % had never previously observed an RFFF. Procedural confidence improved significantly from a median of 2 (IQR 2) to 4 (IQR 1) post-workshop (*p* < 0.001), with greater improvements in those without prior exposure. Anatomical confidence also increased from 3 (IQR 1) to 4 (IQR 2) (*p* < 0.001), particularly among pre-clinical medical students. Participants rated the module highly for educational value, immersion and clarity of anatomical and procedural content.

**Conclusion:**

The VRiMS RFFF teaching module significantly improves learner confidence and anatomical understanding, particularly among early-stage trainees. These findings support the use of VR-based platforms as effective and scalable adjuncts to existing surgical education.

## Introduction

Since its development in 1978, the radial forearm free flap (RFFF) has become a key surgical procedure in head and neck reconstruction. Its popularity stems from having a long vascular pedicle and its anastomosis potential from either the proximal or distal end.^1^ Another strength is its capacity to include composite tissue elements such as vascularized bone, tendon, nerve and muscle, allowing for complex reconstructions.^2^ Due to its thin, pliable nature, the RFFF is particularly suited to the orbital and oral cavity, avoiding the secondary debulking often required with the latissimus dorsi (LD) or anterolateral thigh (ALT) flaps.^3^

Despite its clinical importance, opportunities for medical students and surgical trainees to observe or assist in procedures such as the RFFF remain limited worldwide. Infrequent case exposure, work-hour regulations, service provision demands, and reduced operating theatre access compound the issue.^4^, ^5^, ^6^ In the 2023 Surgical Workforce Census by the Royal College of Surgeons of England (RCSEng), 61 % of surgical trainees identified limited theatre exposure as a key barrier to their development.^6^ These concerns reflect a broader global issue: the World Health Organisation (WHO) and the Lancet Commission on Global Surgery have both highlighted the urgent need to scale up surgical training infrastructure and standardize educational access.^7^^,^^8^ Without strategies to supplement traditional surgical training, the global surgical workforce risks remaining underprepared to meet rising operative demands.

Virtual reality (VR) in surgical training and education has been rapidly expanding within recent years, with numerous specialities harnessing the benefits of this technology to develop trainee skills in a safe and time-efficient manner.^9^ VR surgical training in orthopedics, arthroscopy, and neurosurgery have demonstrated improved skill acquisition, knowledge retention, and operating efficiency compared to traditional methods.^10^, ^11^, ^12^ These benefits have been observed across different VR modalities, including both 360-degree video and six degrees of freedom (6DoF) environments. While both are immersive, 6DoF platforms provide enhanced procedural interactivity, enabling users to manipulate virtual objects and engage in task-specific simulations with spatial accuracy. Whilst VR is being incorporated into various specialities as a means of training residents, there is a continued underrepresentation of this technology within plastic and reconstructive surgery.^13^

The Virtual Reality in Medicine and Surgery (VRiMS) programme is an international educational initiative that utilises immersive extended reality (XR) technology to enhance efficiency and equity in surgical education. This platform facilitates repeated, remote, and standardized exposure to complex surgical procedures for learners at varying levels of training, using 360-degree videos of operations conducted on fresh-frozen cadavers to provide ultra-realistic simulation.

The aim of this study is to evaluate the educational impact of the VRiMS RFFF module at various training levels using indicators such as procedural confidence, anatomical understanding, perceived educational value and qualitative feedback.

## Methods

### Study design

This was a prospective cohort study evaluating the impact of the VRiMS RFFF VR-based training module on the confidence and understanding of the RFFF. This study was reported in accordance with the STROBE (Strengthening the Reporting of Observational Studies in Epidemiology) guidelines.

### Setting and ethical approval

This study was conducted in a controlled simulation environment at 10 medical schools across the United Kingdom (UK) and one NHS trust between 13 April 2024 and 07 April 2025. Each workshop was delivered as a single 60-minute session. Ethical approval for this study was granted by the Brighton and Sussex Medical School (BSMS) Research Governance and Ethics Committee (RGEC), reference number ER/BSMS9GYI/1, in accordance with the Declaration of Helsinki and UK GDPR regulations. Participation was voluntary with participants providing informed consent, after reading the participant information sheet, for their data to be used towards this research study.

### Population and recruitment process

Workshops were open to all UK medical students and healthcare professionals, promoted in coordination with university surgical and medtech societies, and NHS education centers. Participants were recruited through surgical societies, mailing lists and social media platforms.

### Development of the VRiMS RFFF module

A bespoke VR RFFF module was developed as part of the VRiMS programme, designed to deliver immersive surgical education through 360° video and multi-angle recording. The aim was to produce scalable, high-fidelity surgical training resources that simulate real-time operating room environments while allowing participants to rewatch procedures remotely.

### Filming environment and setup

**Figure 1:**
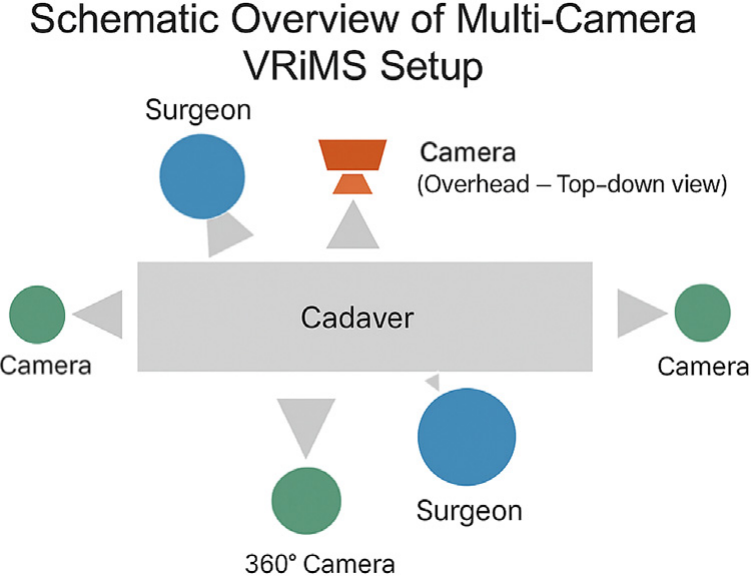
VRiMS multi-camera integration for surgical simulation. Schematic layout illustrates the spatial arrangement of cameras used to capture multiple perspectives of the surgical procedure. A 360° camera is placed for immersive environmental capture. A centrally positioned overhead camera provides a top-down view. Two zoom cameras are positioned to record close-up high-resolution views. Surgeons are stationed at opposing sides of the cadaver, with one surgeon equipped with a head-mounted camera and the other operating a handheld device, enabling dynamic close-up perspectives during procedural demonstrations. This integrated configuration enables synchronized, multi-angle video recording for immersive surgical education.

The procedure was performed on a cadaver at the BSMS anatomy lab, a facility licensed under the UK Human Tissue Act. Recording of the cadaver and faculty participation was performed with fully informed consent. To ensure an immersive educational experience, procedures were recorded using a centrally placed 360° camera. This was supplemented with multiple fixed high-definition (HD) cameras positioned at overhead and lateral angles, as well as surgeon-worn head-mounted cameras and handheld close-up views, providing a composite, multi-perspective visualization of procedural steps (Figure 1).

**Figure 2:**
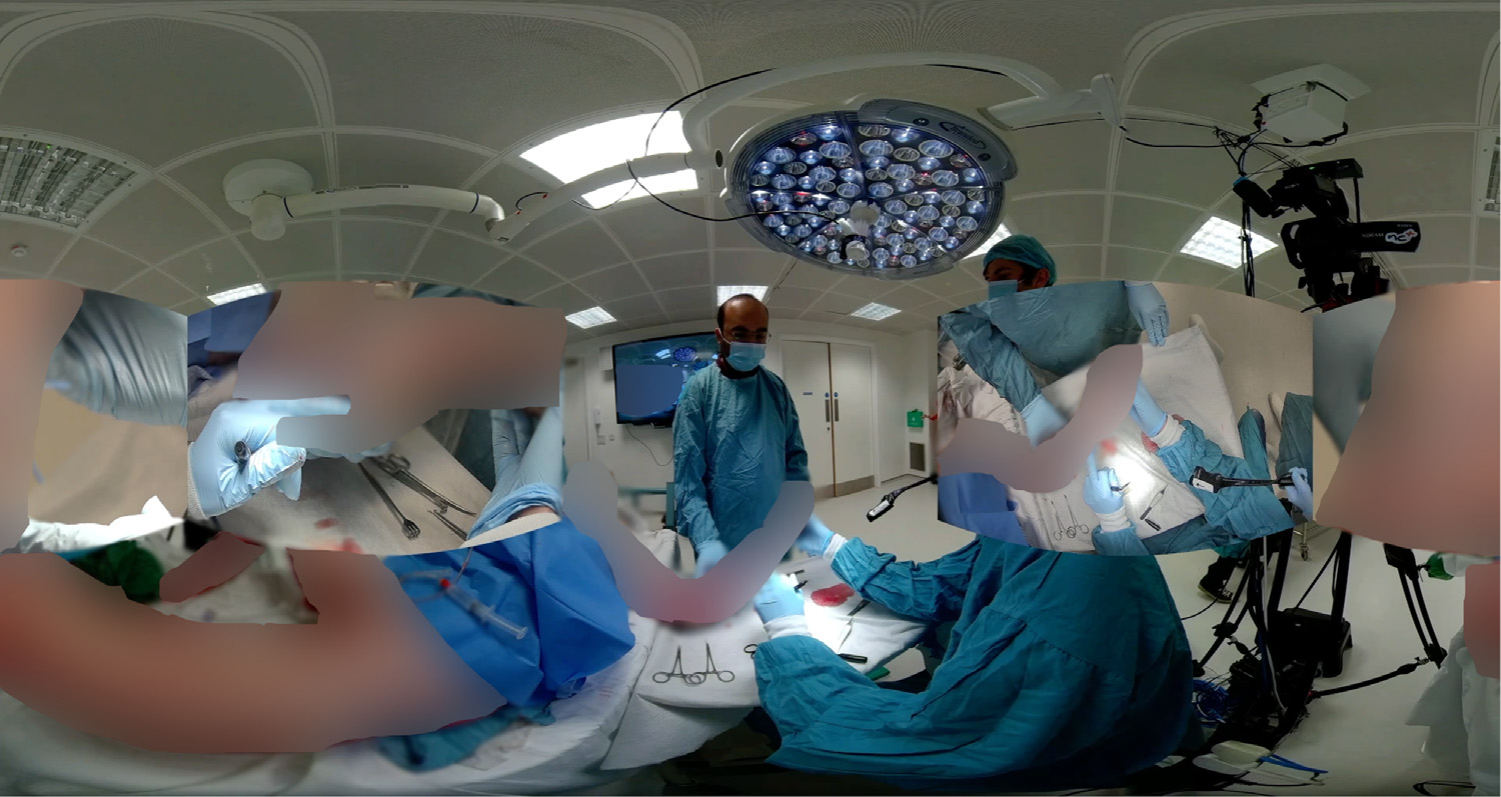
Immersive 360° surgical video capture. Composite view from the VRiMS platform showing a simulated radial forearm free flap procedure. Multiple live camera feeds—including overhead, 360°, and surgeon-held views—are integrated into a single immersive video environment. Cadaveric tissue has been digitally obscured to comply with ethical publication guidelines under the UK Human Tissue Act.

**Figure 3:**
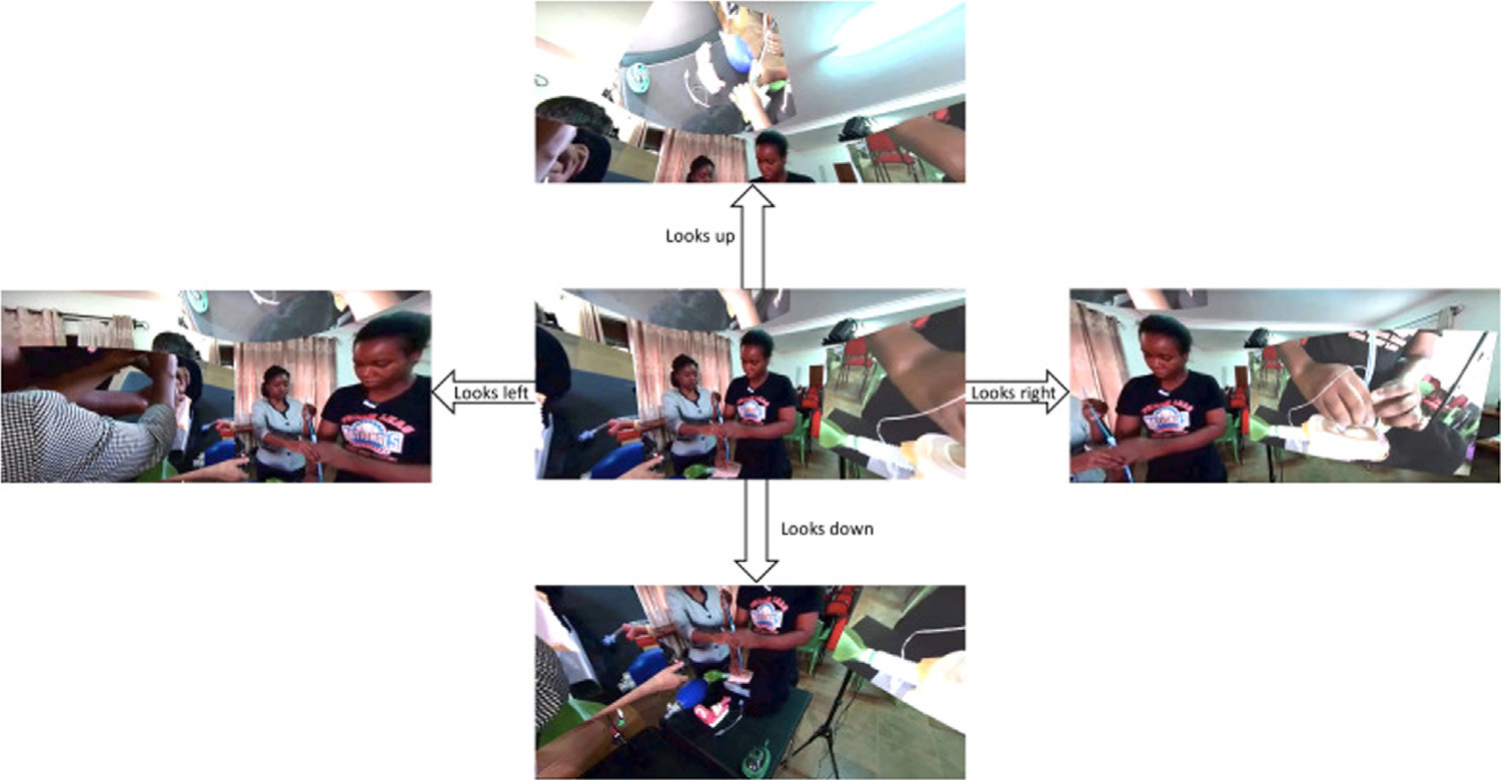
360° surgical video user navigation interface. Example of the user interaction experience within the 360° VR environment. This particular video uses mannequins to demonstrate cricothyroidotomy, allowing unobscured visualization while avoiding restrictions on publishing cadaveric images under the UK Human Tissue Act. The screenshots represent the user’s directional view (up, down, left, right) when navigating the 360° environment via a VR headset. Camera feed overlays are visible in the upper, left, and right views. Reproduced from: Please H, Narang K, Bolton W, et al. *BMJ Open Qual.* 2024;13(1):e002477. Licensed under CC BY-NC 4.0.

The operating setup was organized to mirror a typical surgical field, with all cameras synchronized to enable simultaneous recording and overlay integration. These feeds were processed into a single video file incorporating picture-in-picture views, giving remote participants the ability to observe key anatomical structures and instrument handling techniques from various viewpoints in real time, allowing access to perspectives typically obscured in standard surgical observations (Figure 2). The video also included real-time narration from the lead surgeon performing the procedure. This commentary offered detailed explanations of each surgical step, contextualized within the broader clinical framework. All footage was captured in 5.7 K resolution and stitched using virtual studio software. The resulting media were compatible with a range of devices including: VR headsets, smartphones with gyroscopic control, and laptops and desktops with drag-based navigation (Figure 3).

### Workshop structure

The VRiMS workshop was structured to provide participants with immersive exposure to the RFFF procedure through a two-part VR experience. The workshop was designed to maximize cognitive engagement and anatomical understanding by integrating procedural observation with interactive anatomical exploration. All participants received a pre-workshop setup guide and instructional videos to ensure seamless entry into the virtual learning environment.

The first component of the workshop consisted of watching the VRiMS RFFF procedural simulation delivered through PICO 4 VR headsets. The 360° VR video depicted each stage of the operation in detail, from initial incision planning through to vessel dissection and flap harvest.

**Figure 4:**
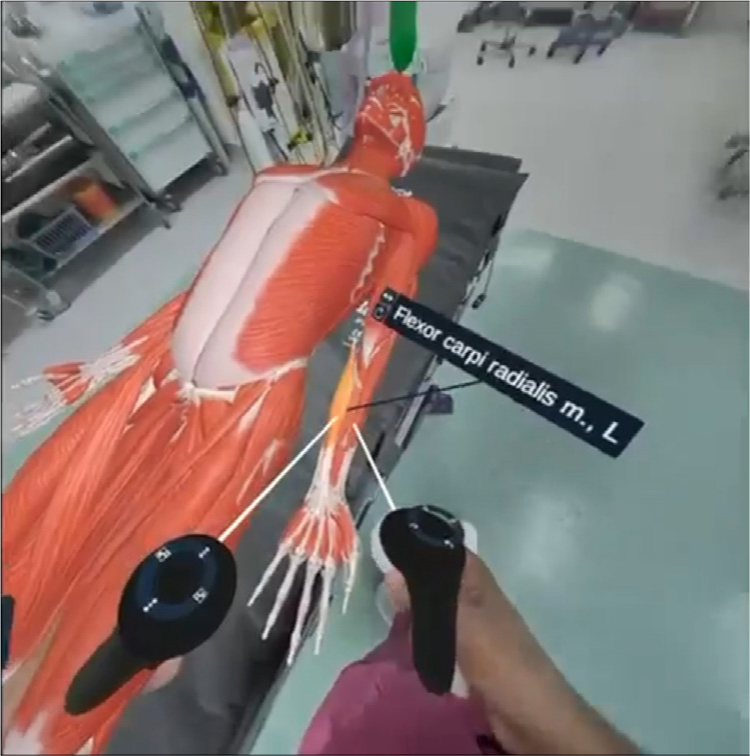
Virtual anatomical dissection of the radial forearm free flap using extended reality. Extended reality-based exploration of the RFFF anatomy using 3D Organon. The model is situated within an operating room to enhance immersion and contextual relevance utilizing pass through capabilities. The flexor carpi radialis muscle is highlighted with interactive labels, facilitating real-time anatomical identification and spatial orientation during flap planning.

The second component focused on interactive anatomical exploration using the 3D Organon™ VR platform. Students engaged with detailed digital models of the forearm in VR, which they could manipulate freely to examine relevant anatomical structures from multiple angles (Figure 4). This session reinforced understanding of the anatomy relevant to the RFFF procedure by allowing participants to explore and visualize the spatial relationships between key structures. Key emphasis was directed towards correlating anatomical knowledge with the surgical procedure, supporting the development of spatial awareness essential for procedural understanding.

### Assessment protocol

A mixed-methods evaluation framework was implemented to assess the educational impact of the VRiMS RFFF module. Participants completed pre- (Appendix A), and post-workshop surveys (Appendix B) via the online platform Google Forms. Outcomes assessed perceived educational value, realism, and immersion of the VR experience, as well as comparison to traditional teaching methods.

The survey instruments utilized a modified version based on the Student Evaluation of Educational Quality (SEEQ) questionnaire originally developed by Marsh (1982).^14^

To further assess the impact of immersion and interactivity within virtual environments, additional items were incorporated from a survey developed by Petersen et al. (2022), which is grounded in the Cognitive Affective Model of Immersive Learning (CAMIL) .^15^ Relevant components from the Teaching Perspectives Inventory (TPI) and the User Experience Questionnaire (UEQ) were also used to assess user engagement.^16^^,^^17^

In addition, questions specifically targeting procedural confidence and perceptions related to the surgical simulation were included. These items utilized a five-point Likert confidence scale, adapted from a validated instrument by Geoffrion et al. (2013), which has been previously used to measure self-efficacy in surgical training contexts.^18^

### Sample size and statistical analysis

A prior sample size calculation was performed using G*Power 3.1. Based on a Wilcoxon signed-rank test for matched pairs, a two-tailed alpha of 0.05, power of 80 %, and an anticipated moderate effect size (Cohen’s *d* = 0.6), the minimum required sample size was determined to be 24 participants. This estimation aligns with sample sizes reported in studies evaluating the impact of virtual reality on surgical education.

Survey responses were initially managed using Microsoft Excel (Version 16.91), and statistical analysis was performed using R (Version 2025.05.0 + 496). Descriptive statistics were used to summarize participant demographics and survey responses. Internal consistency of the Likert-scale items was assessed using Cronbach’s alpha (α = 0.807), indicating good reliability. Normality of continuous data was evaluated using the Shapiro-Wilk test. As data were not normally distributed, non-parametric tests were applied. Paired pre- and post-intervention scores were compared using the Wilcoxon signed-rank test. Differences between groups were assessed using the Kruskal-Wallis test. Likert scale data was summarized using medians and interquartile ranges (IQR). Thematic analysis was used to evaluate qualitative feedback. Statistical significance was defined as *p* < 0.05.

## Results

### Participant demographics

A total of 141 participants completed both pre- and post-workshop assessments. Participants spanned a range of training levels, the majority of participants were medical students (*n* = 128, 90.8 %), comprising 52 pre-clinical (36.9 %) and 69 clinical-year students (48.9 %) with 13 (9.2 %) being postgraduate trainees (F1–registrar level). A complete breakdown of participant training levels are presented in Table 1. Only 13 participants (9.2 %) had previously observed a RFFF procedure during medical training and 37 participants (26.2 %) reported prior experience using VR for medical education purposes.

**Table 1:** Participant training level and prior exposure to the RFFF procedure.

Training level	n (%)	Seen RFFF	Not seen RFFF
Pre-clinical year 1	20 (14.2)	1	19
Pre-clinical year 2	32 (22.7)	3	29
Clinical year 1	31 (22.0)	2	29
Clinical year 2	27 (19.1)	1	26
Clinical year 3	11 (7.8)	1	10
Intercalating	7 (5.0)	0	7
Foundation doctor (F1/F2)	4 (2.8)	0	4
Core trainee (CST/IMT)	4 (2.8)	2	2
Registrar	5 (3.5)	3	2

RFFF, radial forearm free flap. Percentages are calculated based on total *n* = 141.

### Procedural confidence

**Figure 5:**
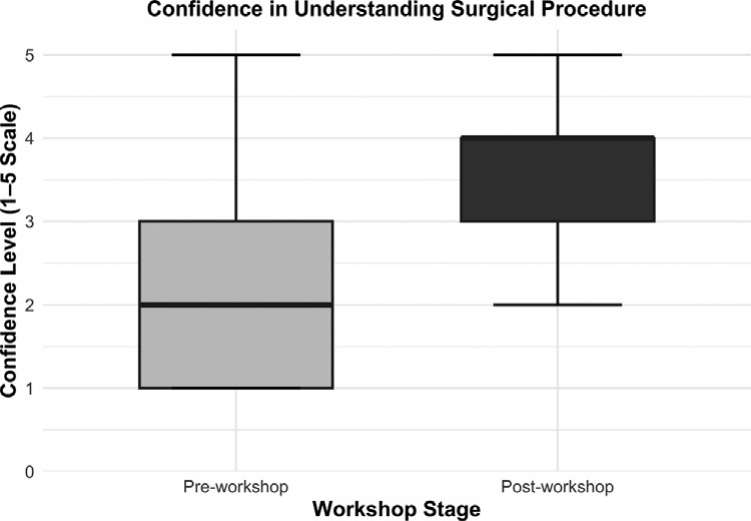
Confidence in understanding surgical procedure. Boxplot comparison of participants’ self-reported confidence in understanding the RFFF procedure pre- and post-workshop. Post-workshop confidence scores (median = 4) were higher than pre-workshop scores (median = 2). Whiskers denote 1.5 × interquartile range.

There was a significant increase in self-reported confidence in understanding the RFFF procedure following the workshop, pre-workshop median: 2 (IQR 2) to post-workshop median: 4 (IQR 1); (*p* < 0.001) (Figure 5). Participants who had not previously seen the procedure demonstrated a greater relative gain in confidence pre: 1 (IQR 1) to post: 4 (IQR 1); (p < 0.001), compared to those who had prior exposure pre: 4 (IQR 2) to post: 4 (IQR 0); (*p* = 0.025).

Stratification by training level demonstrated a statistically significant improvement in procedural confidence among undergraduate participants, with median scores increasing from 2 (IQR 1.25) to 4 (IQR 1); (*p* < 0.001). In contrast, postgraduate trainees showed a smaller but still statistically significant gain, with confidence increasing from a median of 3 (IQR 2) to 4 (IQR 0); (*p* = 0.009). Stratification by individual year group and training stage is presented in Table 2.

**Table 2:** Procedural confidence by training level.

Training level	n	Pre-workshop confidence median (IQR)	Post-workshop confidence median (IQR)	*p*-value
Pre-clinical year 1	20	1 (0.25)	3 (1)	**<0.001**
Pre-clinical year 2	32	1 (1)	4 (1)	**<0.001**
Clinical year 1	31	2 (1)	4 (1)	**<0.001**
Clinical year 2	27	2 (2)	4 (1)	**<0.001**
Clinical year 3	11	1 (2)	3 (1)	**0.013**
Intercalating	7	3 (1)	5 (1)	**0.020**
Foundation doctor (F1/F2)	4	2 (0.5)	3 (0.25)	0.089
Core trainee (CST/IMT)	4	2.5 (3.25)	4 (0)	0.414
Registrar	5	4 (1)	5 (1)	0.089

Median confidence scores pre- and post- VRiMS workshop, with *p*-values from Wilcoxon signed-rank tests. Statistically significant *p*-values are shown in bold.

### Anatomical confidence

**Figure 6:**
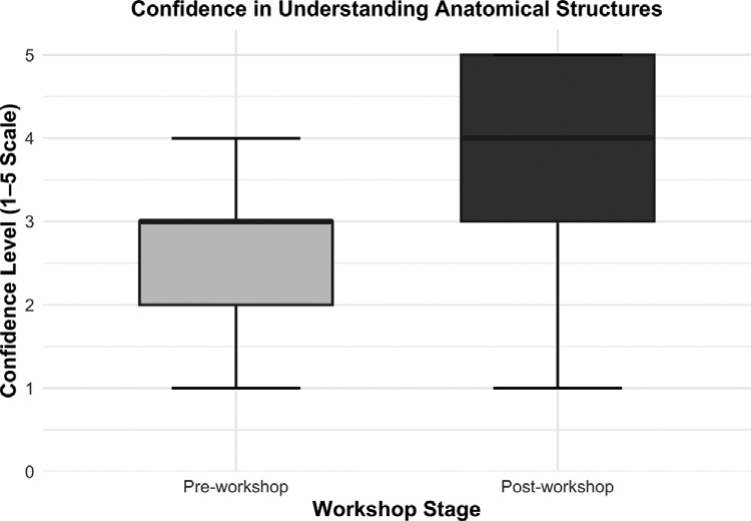
Confidence in understanding anatomical structures. Boxplot comparison of participants’ self-reported confidence in understanding anatomical structures relevant to the RFFF procedure pre- and post-workshop. Median confidence levels increased from pre-workshop (median = 3) to post-workshop (median = 4). Whiskers represent 1.5 × interquartile range.

Participants’ confidence in understanding anatomical structures relevant to the RFFF also significantly improved pre: 3 (IQR 1) to post: 4 (IQR 2); (*p* < 0.001) (Figure 6). Participants who had not previously observed the RFFF procedure demonstrated a similar improvement, with scores increasing from a median of 3 (IQR 1) to 4 (IQR 2); (*p* < 0.001) to those with prior exposure, score change from 3 (IQR 1) to 4 (IQR 2); (*p* = 0.023).

Stratification by training level demonstrated a statistically significant improvement in anatomical confidence among undergraduate participants, with median scores increasing from 2 (IQR 1) to 4 (IQR 2); (*p* < 0.001). In contrast, postgraduate trainees showed a smaller but still statistically significant gain, with confidence increasing from a median of 3 (IQR 1) to 4 (IQR 2); (*p* = 0.040). Stratification by individual year group and training stage is presented in Table 3.

**Table 3:** Anatomical confidence by training level.

Training level	n	Pre-workshop confidence median (IQR)	Post-workshop confidence median (IQR)	*p*-value
Pre-clinical year 1	20	1.5 (1.25)	3 (1.25)	**<0.001**
Pre-clinical year 2	32	3 (1)	4 (0.25)	**<0.001**
Clinical year 1	31	3 (1)	4 (1)	**<0.001**
Clinical year 2	27	2 (1)	4 (2)	**<0.001**
Clinical year 3	11	3 (1)	3 (1)	**0.013**
Intercalating	7	3 (1)	5 (1)	**0.020**
Foundation doctor (F1/F2)	4	3 (0.25)	3 (0.25)	0.089
Core trainee (CST/IMT)	4	3.5 (1.5)	4 (0.5)	0.414
Registrar	5	4 (1)	5 (0)	0.089

Median confidence scores regarding anatomical understanding pre- and post- VRiMS workshop, with *p*-values from Wilcoxon signed-rank tests. Statistically significant *p*-values are shown in bold.

### Perceived educational value of VRiMS

**Figure 7:**
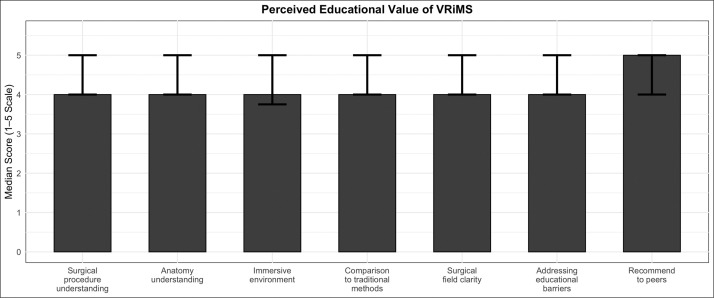
Post-workshop median scores with IQRs across seven VRiMS educational domains. Median post-workshop scores across seven educational domains evaluated after the VRiMS (Virtual Reality in Medicine and Surgery) session. Bars represent median scores on a 1–5 Likert scale, with error bars indicating interquartile range (IQR).

Participants reported high levels of perceived educational benefit across multiple dimensions of the VRiMS experience. The module was rated highly for enhancing understanding of both the surgical procedure (median 4, IQR 1) and the relevant anatomical structures (median 4, IQR 1). The immersive and realistic qualities of the VR experience were similarly well-rated (median 4, IQR 1.25), with many participants noting that it provided a level of engagement and clarity not typically achievable through traditional teaching methods. When compared directly to lectures, textbooks, or hands-on practice, the VR-based session was rated as a more effective method for conveying anatomical and surgical knowledge (median 4, IQR 1). Participants also felt that the session offered a clearer view of the surgical field than they had experienced in clinical theatre environments (median 4, IQR 1), and agreed that it helped address several barriers to surgical education, such as limited theatre space and variability in teaching (median 4, IQR 1). Finally, the majority of participants indicated a high likelihood of recommending the VRiMS workshop to their peers, with the highest possible median score of 5 (IQR 1) (Figure 7). Significant differences were observed across training levels in enhancing understanding of the surgical procedure (*p* = 0.021), perceived immersion (*p* = 0.005) and likelihood of recommending the workshop (*p* = 0.043), with registrars, intercalating students, and senior clinical students rating the experience highest (Table 4).

**Table 4:** Perceived educational value of VRiMS by training level.

Training level	Understanding procedure	Understanding anatomy	Immersion	Compared to traditional methods	Clarity of surgical field	Addressing barriers
Pre-clinical year 1	4 (1)	4 (1)	3 (1)	5 (1)	4 (2)	4 (1.5)
Pre-clinical year 2	4 (1)	4 (1)	4 (1)	4 (0.5)	4 (1)	4 (1)
Clinical year 1	4 (1)	4 (1)	4 (1)	4 (1)	4 (1)	4 (1)
Clinical year 2	4 (2)	4 (1)	4.5 (1)	4 (1)	4.5 (1)	4 (1)
Clinical year 3	3 (1)	4 (1)	4 (1.5)	4 (1)	4 (1.75)	4 (1)
Intercalating	5 (1)	5 (1)	4 (0)	4 (0)	4 (0)	4 (0)
Foundation doctor (F1/F2)	3 (0.5)	3 (0.5)	3 (0.5)	3.5 (1.25)	3 (0.25)	3.5 (1.25)
Core trainee (CST/IMT)	4.5 (1)	4.5 (1)	4.5 (1)	4 (0.5)	4.5 (1)	4.5 (1)
Registrar	5 (1)	5 (1)	5 (0)	5 (0)	5 (0)	5 (0)

Participant ratings of the VRiMS module across key educational dimensions. Values are presented as median (IQR). Significant differences across training levels were observed in enhancing understanding of the surgical procedure (*p* = 0.021) and immersion (*p* = 0.005).

### Qualitative feedback

Open-ended responses highlighted several perceived strengths of the VRiMS module. Participants frequently cited the value of the first-person surgical perspective, the ability to explore detailed anatomy interactively, and the immersive sensation of “being in theatre” as unique educational benefits. The 360° VR environment with multiple overlays was particularly appreciated for allowing unrestricted visual access to procedural steps and anatomical landmarks that are often difficult to observe in conventional teaching formats. Suggestions for improvement focused on technical and structural aspects of the experience. Several participants noted occasional challenges with headset clarity and suggested enhancements in image resolution and adjustable interpupillary distance. Others recommended extending the duration of the session to allow more time for the VR experience.

## Discussion

This multicenter prospective study demonstrates that the VRiMS workshop of the RFFF significantly improves participants’ confidence in both anatomical understanding and procedural knowledge. These findings highlight the potential of VR as a scalable and effective adjunct to traditional surgical education.

### Benefits

Our participant data (*n* = 141) identified several systemic challenges in current surgical education: limited procedural exposure (80.7 %), restricted theatre space (71.6 %), and variability in teaching quality (68.2 %). Notably, 90.8 % of participants had never observed an RFFF procedure before. These findings are consistent with national reviews of surgical training in the UK, where medical students and resident doctors report theatre time as unstructured, disempowering, and lacking in educational value. Many describe being unable to see procedures, unclear on their role, and discouraged from asking questions.^5^^,^^19^, ^20^, ^21^ Within current surgical training, traditional “see one, do one, teach one” apprenticeship models are increasingly constrained by reduced trainee working hours, theatre scheduling and the need for adequate operative volume^4^ and patient safety concerns.^22^, ^23^, ^24^

VRiMS addresses this by providing an immersive, standardized, and repeatable training tool. This transforms surgical learning from an opportunistic, passive experience into a structured educational environment. The inclusion of live commentary, within the simulation, from the operating surgeon ensures that every learner receives expert-led, step-by-step procedural instruction. Crucially, the VRiMS simulation incorporates multi-angle camera overlays, including head-mounted, overhead, and close-up intraoperative views. This allows learners to experience perspectives that are often physically inaccessible in crowded theatres. This ensures that learners can appreciate intricate technical details, overcoming the spatial and logistical constraints of live theatre teaching. Repeatability further strengthens VRiMS’ educational impact. Learners can pause, rewind, and rewatch procedural content multiple times, promoting spaced repetition and remote learning. This approach is known to improve long-term retention with users trained through VR being up to 16 times more likely to recall information than those trained via traditional methods.^25^

VRiMS also offers clear advantages over 2D video formats. Unlike passive recordings, immersive VR allows learners to control their field of view, explore anatomical detail from different angles, and actively engage with the environment. Studies have shown learners utilizing VR are up to 3.75 times more emotionally connected to content, 4 times more focused, and 275 % more confident applying skills learned through VR.^26^ Studies looking at surgical training demonstrated that VR-trained participants achieved up to 300 % higher proficiency scores and made 67 % fewer errors than standard groups.^27^^,^^28^

### Curricular relevance

The recent expansion of medical school places in the UK further highlights the need for scalable educational tools.^29^ With variable theatre access across institutions, many students risk graduating without exposure to key surgical techniques.^30^ VRiMS addresses this disparity by enabling learners to access complex procedural content—regardless of geography, scheduling, or case availability. The Royal College of Surgeons of England’s (RCSEng) Future of Surgery Commission has endorsed extended reality as a core component of future surgical education, citing its potential to enhance rehearsal, reduce error, and democratize training access.^31^ Integration of VR-based programmes like VRiMS into mainstream curricula aligns directly with these national recommendations.

### Global health

The applicability of VR-based surgical education is particularly evident in low-resource settings, where access to operative exposure and mentorship remains limited. The Surgical Theatre Educational Environment Measure (STEEM) assesses perceptions of the operating theatre as a learning environment among medical students and surgical trainees.^32^^,^^33^ A study conducted in Nigeria revealed that only 38 % of trainees rated their theatre-based education as satisfactory, citing limited case variety as a contributing factor.^34^ Similar findings have been reported in Sudan, where STEEM scores reflected concerns over the volume and quality of elective procedures available for educational purposes.^35^ The affordability and portability of standalone VR headsets create a unique opportunity to deliver standardized, accessible, high-quality surgical education across global health settings.^36^, ^37^, ^38^

## Limitations

This study has several limitations. It was conducted at a single time point without follow-up to assess long-term knowledge retention or skill transfer to clinical practice. Confidence scores reflect self-perception rather than technical competence or patient outcomes, although prior research supports their validity in simulation-based education.^39^^,^^40^ This study also lacked randomization or a control group. Additionally, the VRiMS module does not include haptic feedback or psychomotor training, limiting its role in manual skill development; however, this may be less critical for early-stage learners focused on cognitive and visual learning.^41^ Furthermore, subgroup analyses, particularly among individual postgraduate trainee levels, may have been underpowered due to small sample sizes.

### Future directions

Future research should focus on longitudinal studies assessing knowledge retention, clinical translation and performance outcomes following VR-based training. Randomized controlled trials using validated assessment tools, such as OSATS, are needed to compare VR modules with conventional teaching methods and establish their educational value. Further validation of VRiMS across a broader range of procedures and surgical trainee levels will help determine its role within surgical curricula. Future studies will also assess the integration of supplementary software enabling synchronized and interactive VR experiences, which may further enhance engagement and collaborative learning. Finally, evaluating the feasibility, cost-effectiveness and educational impact of VR-based training in low-resource settings will be important for informing its global application.

## Conclusion

This multicenter prospective study found that the VRiMS RFFF module significantly improved self-reported confidence in procedural steps and anatomical understanding, particularly among pre-clinical and early-stage surgical trainees. Participants rated the module highly in terms of educational value, realism, and engagement. These findings support the use of virtual reality-based platforms as effective, scalable adjuncts to traditional surgical education, with potential to address variability in operative exposure and standardize early procedural training.

## Role of the funding source

This research was supported by the ASiT Surgical Research Training Grant 2024. The funding body had no role in the design, data collection, analysis, interpretation, or writing of the manuscript.

## Ethical approval

Ethical approval for this study was granted by the Brighton and Sussex Medical School Research Governance and Ethics Committee (RGEC), reference number ER/BSMS9GYI/1.

## Declaration of competing interest

Professor Jag Dhanda is the CEO of VRiMS, the platform used for the educational intervention in this study, and served as the academic supervisor for this project. He was not involved in data collection or statistical analysis. All other authors declare no conflicts of interests.

## Appendix Supplementary materials

Image, application 1

Image, application 2
