# Supplementary material for: Development and impact of virtual reality-based training for the radial forearm free flap: A multi-center prospective feasibility study
Source: JPRAS Open. 2025 Oct 24;48:65–79. doi: 10.1016/j.jpra.2025.10.024 (PMC12686927; doi:10.1016/j.jpra.2025.10.024)
Supplement: Supplementary file 1 [file mmc1.docx]

**Annex**

**Appendix A.** Pre-Workshop Survey

1. What training level are you?

- Medical Student: Preclinical Year 1
- Medical Student: Preclinical Year 2
- Medical Student: Clinical Year 1
- Medical Student: Clinical Year 2
- Medical Student: Clinical Year 3
- F1/F2
- Core Trainee (CST/IMT)
- Registrar
- Consultant
- Other

1. Have you used a VR headset in the context of medical education before this session?

- Yes
- No

1. How relevant do you believe is extended reality technology in the future of medical education?

- Scale: 1 (Not Relevant) to 5 (Highly Relevant)

1. What do you think are the current barrier(s) to surgical education? (Tick all relevant)

- Limited theatre space
- Breadth of surgical knowledge/skills required
- Limited exposure to a variety of surgical procedures
- Variability in quality of mentorship and training
- Insufficient focus on anatomical understanding in current training programmes
- Other

1. Have you ever seen a radial forearm free flap before during your formal medical training?

- Yes
- No

1. How confident are you in your understanding of a radial forearm free flap?

- Scale: 1 (Not Confident) to 5 (Very Confident)

1. How confident are you in your understanding of anatomical structures relevant to a radial forearm free flap?

- Scale: 1 (Not Confident) to 5 (Very Confident)
