# Supplementary material for: Development and impact of virtual reality-based training for the radial forearm free flap: A multi-center prospective feasibility study
Source: JPRAS Open. 2025 Oct 24;48:65–79. doi: 10.1016/j.jpra.2025.10.024 (PMC12686927; doi:10.1016/j.jpra.2025.10.024)
Supplement: Supplementary file 2 [file mmc2.docx]

**Appendix B.** Post-Workshop Survey

1. How confident are you in your understanding of a radial forearm free flap after the VRiMS workshop?

- Scale: 1 (Not Confident) to 5 (Very Confident)

1. How confident are you in your understanding of anatomical structures relevant to a radial forearm free flap after the VRiMS workshop?

- Scale: 1 (Not Confident) to 5 (Very Confident)

1. To what extent did the VR experience enhance your understanding of a radial forearm free flap?

- Scale: 1 (Not at All) to 5 (Greatly Enhanced)

1. To what extent did the VR experience enhance your understanding of anatomical structures relevant to a radial forearm free flap?

- Scale: 1 (Not at All) to 5 (Greatly Enhanced)

1. How realistic and immersive did you find the VR experience in terms of replicating a real clinical/surgical environment?

- Scale: 1 (Not at All) to 5 (Very Realistic)

1. How does the VR session compare to traditional learning methods (e.g., lectures, textbooks, hands-on practice) in helping you understand anatomy and surgery?

- Scale: 1 (Much Worse) to 5 (Much Better)

1. How well does the VR session provide a clear view of the surgical field compared to your experience in the operating theatre?

- Scale: 1 (Much Worse) to 5 (Much Better)

1. To what extent did the VRiMS session address current barriers to surgical education?

- Scale: 1 (Much Worse) to 5 (Much Better)

1. How likely are you to recommend the VRiMS workshop to fellow colleagues?

- Scale: 1 (Very Unlikely) to 5 (Very Likely)

1. What was the most valuable part of the workshop for you? (Optional)

- Open-ended

1. Do you have any suggestions for improving the VRiMS workshop experience? (Optional)

- Open-ended

1. Do you consent to your data being used for research purposes?

- Yes / No
